# Contribution of prokaryotes and eukaryotes to CO_2_ emissions in the wastewater treatment process

**DOI:** 10.7717/peerj.9325

**Published:** 2020-06-17

**Authors:** Katarzyna Jaromin-Gleń, Roman Babko, Tatiana Kuzmina, Yaroslav Danko, Grzegorz Łagód, Cezary Polakowski, Joanna Szulżyk-Cieplak, Andrzej Bieganowski

**Affiliations:** 1 Institute of Agrophysics, Polish Academy of Sciences, Lublin, Poland; 2 Schmalhausen Institute of Zoology, National Academy of Sciences, Kiev, Ukraine; 3 Sumy State University, Sumy, Ukraine; 4 Sumy Makarenko State Pedagogical University, Sumy, Ukraine; 5 Environmental Engineering Faculty, Lublin University of Technology, Lublin, Poland; 6 Fundamentals of Technology Faculty, Lublin University of Technology, Lublin, Poland

**Keywords:** CO_2_ emission, Eukaryotes, Prokaryotes, Sequencing batch reactor

## Abstract

Reduction of the greenhouse effect is primarily associated with the reduction of greenhouse gas (GHG) emissions. Carbon dioxide (CO_2_) is one of the gases that increases the greenhouse effect - it is responsible for about half of the greenhouse effect. Significant sources of CO_2_ are wastewater treatment plants (WWTPs) and waste management, with about 3% contribution to global emissions. CO_2_ is produced mainly in the aerobic stage of wastewater purification and is a consequence of activated sludge activity. Although the roles of activated sludge components in the purification process have been studied quite well, their quantitative contribution to CO_2_ emissions is still unknown. The emission of CO_2_ caused by prokaryotes and eukaryotes over the course of a year (taking into account subsequent seasons) in model sequencing batch reactors (SBR) is presented in this study. In this work, for the first time, we aimed to quantify this contribution of eukaryotic organisms to total CO_2_ emissions during the WWTP process. It is of the order of several or more ppm. The contribution of CO_2_ produced by different components of activated sludge in WWTPs can improve estimation of the emissions of GHGs in this area of human activity.

## Introduction

When considering carbon dioxide (CO_2_) production in the wastewater treatment process, a distinction should be made between indirect and direct emissions (Massara et al., 2017). The energy consumption of devices working in the wastewater treatment plant (WWTP) causes the indirect emissions. All these devices require an electric power supply. Most WWTPs use electric motors for screen cleaning systems and transport of screenings and sand/gravel from grit chambers, sludge scrapers in primary and secondary settling tanks, drive of sludge treatment devices and above all, supplying system blowers for the aeration of oxygen zones within bioreactors (USEPA, 2013; Bao, Sun & Sun, 2015; Drewnowski et al., 2019). Direct emissions are caused by organisms found in the wastewater purification process. There are many groups of organisms that take part in this process, however, taking into account that popular technologies are based on activated sludge in the form of flocs, mainly prokaryotes and eukaryotes can be considered (Jaromin-Gleń et al., 2013; Kong et al., 2016). Wastewater treatment—regardless of the technology used – causes CO_2_ emissions. However, the largest amounts of this gas are released into the atmosphere during the aerobic phase. This is connected with the respiration of both the prokaryotes and eukaryotes present in activated sludge during the decomposition of organic matter in the wastewater. During this process, prokaryotes are mainly involved in removing carbon (C) compounds and biogenic substances in dissolved forms, whereas eukaryotes remove bigger suspended particles—also not flocculated prokaryotes. Eukaryotes collect (take up) suspended particles, decompose them and incorporate them into their own biomass; in this way, eukaryotes cause removal, decomposition and transformation of pollution. Eukaryotes, in addition to removing suspended particles, actively (mostly by grazing) prey on living within and on flock prokaryotes (flocculated ones). Eukaryotes, therefore, exert ecological pressure on prokaryotes and stimulate them to grow, helping them to form flocks of proper size, shape and density parameters, indispensable for proper sedimentation. The above-mentioned ecological pressure and the removal of suspension are the main tasks of eukaryotes in a normally functioning activated sludge in the formation of flocks. During these biological activities, eukaryotes emit CO_2_ (Čech, Hartman & Macek, 1994; Pajdak-Stós et al., 2010; Kong et al., 2016; Babko et al., 2017)_._

Sequencing batch reactors (SBRs) are not as popular as flow treatment plants, however, the lower investment cost of a single reactor and greater operational flexibility, makes SBR technology a reasonable alternative in every scale of WWTP (Shingh & Srivastava, 2011). They are often used for industry factories (for instance in food industry), housing estates or small towns (Fernandes et al., 2013; Quan & Gogina, 2019).

From a biochemical point of view, processes occurring at flow-mode and sequencing reactors with activated sludge are similar. The main difference is that in the flow system, the subsequent processes occur in a series of chambers and in SBRs aerobic, anoxic and anaerobic processes are carried out in one chamber. Therefore, the SBR wastewater treatment process has the following successive stages: (i) filling the chamber, (ii) mixing (the anoxic and anaerobic processes start at this stage), (iii) aeration (intensification of the aerobic processes), (iv) settling (sedimentation of activated sludge, when at this same time the aerobic, anoxic and sometimes anaerobic processes occur), (v) decantation and (vi) idle phase (smooth mixing to condition the biomass and sludge wasting). The consequence of different ways of wastewater treatment is different dynamics and amount of gas emissions.

While it is known that activated sludge microorganisms (prokaryotes and eukaryotes) carry out the decomposition of organic substances—and prokaryotes are responsible for most of this process—to the best of the authors’ knowledge, there is no research identifying how total emissions of CO_2_ are divided, that is, estimating the parts emitted by prokaryotes and eukaryotes. The aim of this work is to determine the quantitative contribution in emissions of CO_2_ by each group. The emission of CO_2_ caused by prokaryotes and eukaryotes over the course of a year (taking into account seasonal differences) in a laboratory-scale model SBR is presented in this study. Use of a small scale enabled us to accurately investigate the CO_2_ emission, because the space above the wastewater in a full-scale WWTP is open, which causes a problem with the representative and reproducible sampling of gases emitted from the reactor.

## Materials and Methods

### Model of SBR bioreactors

The laboratory model consisted of three independent SBR chambers/reactors with a total volume of 2 dm^3^ and an active volume of 1.8 dm^3^ of each. Each reactor was equipped with a mechanical stirrer, an aeration system with a membrane diffuser and a temperature stabilization system.

A full SBR operation cycle was set to 12 h and was characterized by the following phases: (i) filling the chamber (10 min), (ii) mixing (180 min), (iii) aeration (420 min), (iv) settling (90 min), (v) decantation (10 min) and (vi) the idle phase (10 min). The selection criterion for the duration of the cycle phases was the effectiveness of wastewater purification checked during the preliminary investigations.

The aeration phase (420 min) was divided into sub-phases: (1) aeration (150 min), (2) break (15 min), (3) aeration (15 min), (4) break (15 min), (5) aeration (15 min), (6) break (15 min), (7) aeration (15 min), (8) break (30 min), (9) aeration (15 min), (10) break (30 min), (11) aeration (15 min), (12) break (30 min), (13) aeration (15 min), (14) break (30 min), (15) aeration (15 min).

To take into account seasonal changes concerning both the conditions of the purification process and the quality of the wastewater, the experiment was divided into 4 seasons–4 experiments, each with an individual time schedule. The temperature of the reactors for each season was set at a level (±1 °C) recorded as the average temperature in the municipal WWTP in Lublin (south-east of Poland): that is, spring and autumn: 15 °C; winter: 10 °C and summer: 20 °C. To simulate real conditions as closely as possible and to improve the reliability and research value of the experiment, raw, city wastewater was used. To minimize time and cost of logistics, the closest WWTP (Lublin, Poland) was selected for regular wastewater collection. From a biochemical point of view, the processes carried out in the Lublin full-scale WWTP and the laboratory experiment were the same—the combined removal of C, nitrogen (N) and phosphorous (P) was carried out in alternating anoxic, anaerobic and aerobic conditions using the activated sludge method. This meant that, nitrification and denitrification, as well as biological removal of P and C were carried out. Hence, the activated sludge from the Lublin WWTP could be used for inoculation in our SBR.

During the biological removal of N compounds, nitrification occurs under aerobic conditions, the end effect of which is the production of nitrates. These nitrates in turn are transformed under anoxic conditions and removed from the system into the atmosphere in the form of molecular N. At subsequent SBR work cycles, nitrates needed at the anoxic stage are therefore the product of the previous aerobic stage. At the start of the SBR operation, there are no nitrates from previous cycles, so they must be supplied externally.

To ensure an accurate representation of the activated sludge and raw wastewater, the samples were collected around 1 month after the beginning of the season: that is, the experiment in the spring season started on 15th April, in summer—on 15th August, in autumn—on 15th October and in winter—on 15th January.

The startup mixtures in the SBR laboratory reactors were: (i) activated sludge as a process factor (0.6 dm^3^), (ii) raw wastewater (0.5 dm^3^), (iii) wastewater after purification as a source of nitrates (0.7 dm^3^). A total of 0.5 dm^3^ of purified wastewater was removed from the SBR during the decantation phase in each cycle. The same amount (i.e., 0.5 dm^3^) of raw wastewater was added during the filling phase. The above volumes were adjusted to the capacity of our SBR, but the proportions represent typical ranges used in laboratory bioreactors (Babko et al., 2015; Sytek-Szmeichel, Podedworna & Zubrowska-Sudol, 2016; Alzate Marin, Caravelli & Zaritzky, 2016; Yao et al., 2019).

The effectiveness and stability (stability understood as maintaining adequate purification effectiveness over time) was monitored with the use of physicochemical indicators: dissolved oxygen, redox potential, pH, turbidity, and content of total suspended solids (TSS). Dissolved oxygen was monitored online using a DO meter (probe LDO; Hach, Loveland, CO, USA) working with a control station (SN 100; Hach, Loveland, CO, USA). To obtain the efficient nitrification and biological oxidation of C compounds, the parameter was established between 2.0 and 2.5 mg O_2_∙dm^−3^. pH and redox potential in the reactors were monitored online with a multimeter (HQ 440D; Hach, Loveland, CO, USA). The observation of these parameters enabled evaluation of the smooth running of the process. The effectiveness of removing pollutants in the form of suspended solids and the filtering work of eukaryotic organisms, monitored by chemical oxygen demand (COD) were determined spectrophotometrically by means of a Hach DR 3900 spectrophotometer, using the relevant reagents, according to the method recommended by the manufacturer. TSS were determined spectrophotometrically (DR 3900 Benchtop VIS, Hach, Loveland, CO, USA) according to the standard method provided by the producer. Turbidity was measured using a turbidimeter (CyberScan TN 100; Eutech Instruments, India) in accordance with the instructions provided by the manufacturer.

### Identification of eukaryotic microorganisms

The identification and determination of the species composition and the calculation of the numbers of eukaryotic organisms were performed using a transmitted light mode optical microscope (CX41; Olympus, Tokyo, Japan). The phase contrast or the dark-field method was used when needed. The samples were counted under an 18 mm × 18 mm cover glass. One subsample had a volume of 25 cm^3^. Microscopic examinations of the samples were performed in vivo in five replications. The eucaryotes were counted immediately after activated sludge sampling, (approx. 30 min after aeration start—this time was sufficient to fully mix the activated sludge with wastewater). Several taxonomic guides (Kahl, 1930; Foissner et al., 1991, 1995; Foissner, Berger & Kohmann, 1992, 1994; Foissner & Berger, 1996; Serrano, 2008; Berger & Foissner, 2014) were used for ciliate species identification. The results were averaged and recalculated to 1 cm^3^. Sample processing methodology, eukaryotes determining and counting was described by (Babko et al., 2017).

### Gas analysis

The concentration of CO_2_ in the SBR above the wastewater surface was measured during the aeration phase for several reasons:

- Aerobic respiration of activated sludge organisms and CO_2_ formation took place in this phase.
- The emission in the other phases was so low that it was practically impossible to measure the fluxes of gases; as a consequence, the error of flux measurement would be very great.
- The gases (not only CO_2_) produced during the other phases were trapped physically in the activated sludge volume. This additionally reduced the amount of gas released from the volume of the bioreactor. These gases were released partly during mixing and, primarily, during aeration. Better efficiency of gas release during aeration was achieved by better mixing of the activated sludge with passing air bubbles, rather than by the mechanical stirrer. Thus, the gas analysis during aeration also allowed partial consideration of gaseous emissions in the other phases.

As the full SBR operation cycle was set to 12 h, there were two cycles a day. Gas samples were collected from each chamber during the first cycle each day. Sampling was carried out 8 times in the aeration phase: at 5 min, 15 min, 75 min, 135 min (aeration), 157 min (break in aeration), 172 min (aeration), 187 min (break in aeration) and 202 min (aeration). The gas was collected from the reactor headspace with a syringe (25 cm^3^ volume) attached to a hose. The gas sample collected was transferred to a 20 cm^3^ glass vial, submerged and filled with water. The filling of the vial with the gas sample caused the removal of water from the vial—allowing the vial to be filled only by gas from the experiment and not by air from the laboratory. Vials were closed with a rubber stopper, removed from the water, sealed with an aluminum cap and placed in a gas chromatograph autosampler tray.

GC-2014 Shimadzu with a PLOT Supel-Q column FID detector and autosampler was used to measure CO_2_ concentration. It was assumed that the amount of gases entering the chamber in the aeration phase equalled the amount of gases coming out of the chamber. The flow rates were read from rotameters (400 cm^3^·min^−1^).

The CO_2_ flux density emitted was calculated based on the Eq. (1) (Liu et al., 2014)

(1) $$E_{{\rm total\; CO}_2} = {\rm \rho} \cdot c\cdot \displaystyle{Q \over A}$$

where:

*E*, total CO_2_ flux density (g·m^−2^·min^−1^).

ρ, gas density at a given temperature (g·dm^−3^).

*c*, CO_2_ concentration expressed by the dimensionless volume fraction.

*Q*, all gases flux (dm^3^·min^−1^).

*A*, surface of the reactor chamber (m^2^).

### Calculation of CO_2_ emissions caused by eukaryotes and prokaryotes

Assuming that the amount of CO_2_ released into the atmosphere is equivalent to the amount of oxygen used in the respiration process, the formula (Eq. (2)) proposed by (Kovalchuk & Babko, 1997) was used. Coefficient values take into account the experimental conditions used:

(2) $${R_{\rm eucaryota}} = {34.217} \cdot W_{\rm m}^{0.672}\cdot 1.667\cdot {10^{ - 8}}$$

where:

*R*, respiration rate, which is biomass CO_2_ emission rate at 20 °C (dm^3^·min^−1^).

*W*_m_, mass of the eukaryotic organisms (ng).

1.667·10^−8^, µl·h^−1^ (as in (Kovalchuk & Babko, 1997)) to dm^3^·min^−1^ conversion coefficient.

Since the actual temperatures in the SBR in the autumn, winter and spring were different from 20 °C, the values calculated on the basis of Eq. (2) were corrected by coefficients (Winberg, 1983) *Q*_15 °C_ = 1.5 and *Q*_10 °C_ = 2.25.

*W*_m_ was the average weight of the individual, tested species, multiplied by their number. The average weights of individuals from each species were taken from the literature (Foissner et al., 1991, 1995; Foissner, Berger & Kohmann, 1992, 1994; Foissner & Berger, 1996).

The eucaryota CO_2_ emission was then calculated as follows:

(3) $${E_{\rm eucaryota}}= \displaystyle{{{R_{\rm eucaryota}}\cdot M} \over {V\cdot A}}$$

where:

*E*_eucaryota_, total emission of eucaryota (g·m^−2^·min^−1^).

*M*, molar mass of CO_2_, which is 44 g·mol^−1^.

*V*, volume of 1 mol of CO_2_ at a given temperature (23.23 dm^3^ in 10 °C; 23.63 dm^3^ in 15 °C; 24.04 dm^3^ in 20 °C).

*A*, area of SBR water surface (0.005 m^2^).

As we assume that the main factor responsible for wastewater purification is prokaryote (Wagner & Loy, 2002; Fernandes et al., 2013; Zhang et al., 2019) CO_2_ emissions caused by prokaryotic organisms were calculated as the difference between the total amount of CO_2_ (the total emission obtained by GC) and the amount produced by the eukaryotic organisms as follows:

(4) $$E_{\rm procaryota} = E_{\rm total\; CO_{2}} - E_{\rm eucaryota}$$

### Statistical analysis

Carbon dioxide emission data were analysed statistically using STATISTICA 12. The statistical significance of the differences between the seasons was analysed by one-way analysis of variance and Tukey’s post-hoc tests.

## Results

All parameters measured, demonstrated that the process of wastewater purification was lasting and effective. pH was stable and was in the range of 7.5–8.5. DO was kept within the assumed scope that is, 2.0 and 2.5 mg O_2_∙dm^−3^. Redox potential during the aeration phase was in the range of −150 mV to +200 mV. COD of purified wastewater was between 10 and 25 mg∙dm^−3^, while total suspending solids (TSS) were 0.5–5.0 mg∙dm^−3^ and turbidity was 1.0–4.0 NTU.

A total of 32 taxa of ciliates (10 crawling, 17 attached and five free-swimming), two taxa of testate amoebas, two taxa of naked amoebas, one taxon of flagellates, five taxa of rotifers and one taxon of nematodes were identified through composition analysis of the activated sludge. The greatest abundance of eukaryotic species occurred in the spring and summer period (25 species) and the smallest number was recorded in the autumn (19 species). However, the largest number of individuals was noted during the autumn period—5472 (SD = 419) indiv∙cm^−3^ and the smallest number was found in the spring—1,688 (SD = 40) indiv∙cm^−3^. Thus, the qualitative composition of eukaryotic organisms in experiments had statistically significant seasonal differences (*p* ≤ 0.05).

**Figure 1:**
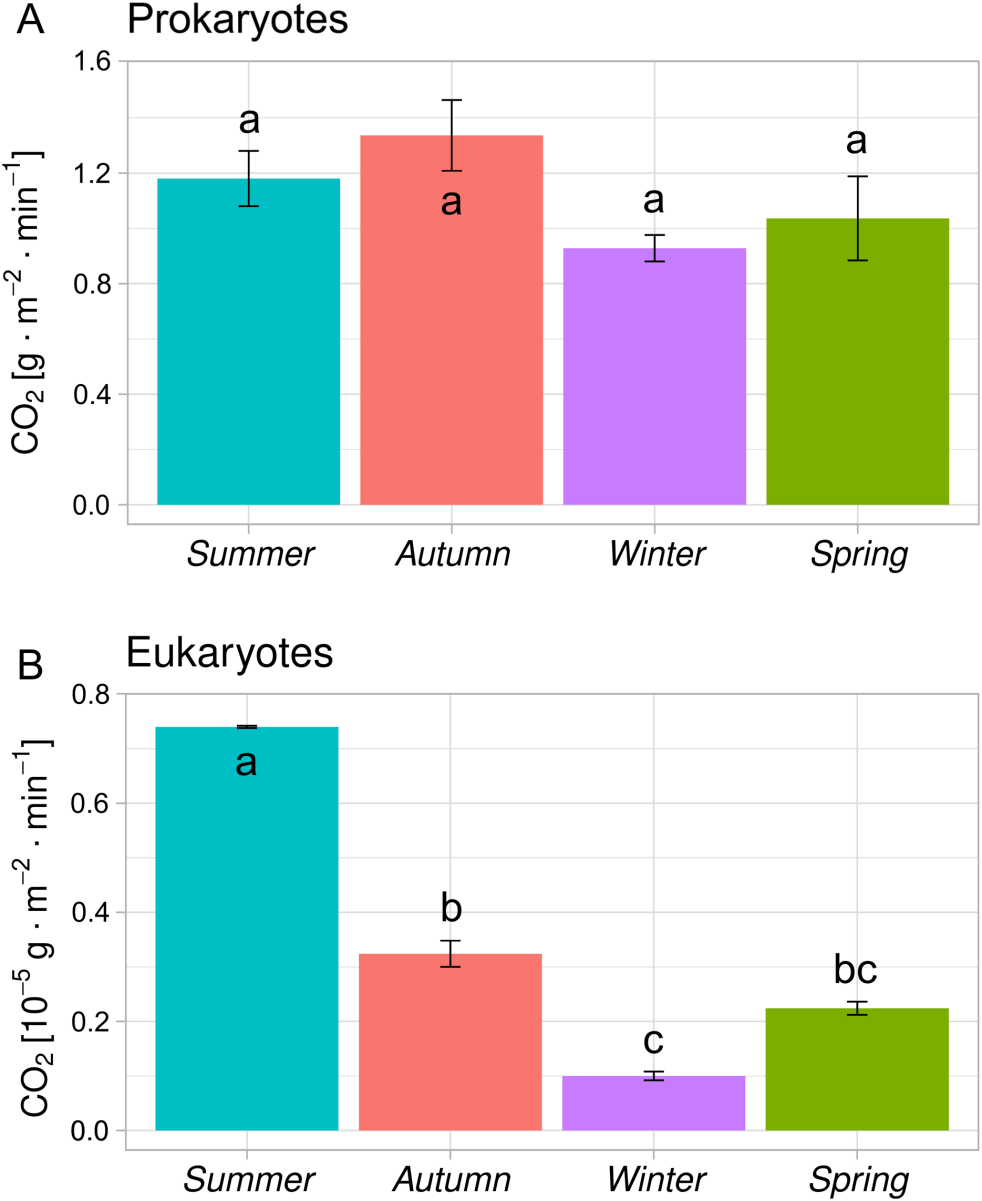
Averaged emission of CO_2_ caused by the activity of prokaryotes and eukaryotes (*n* = 24). The whiskers show standard deviation. The lowercase letters indicate the significance of the differences between the bars (post-hoc Tukey HSD test, *p* < 0.05).

In order to assess the intensity of oxygen consumption and, accordingly, the emission of CO_2_ by ciliated protozoa, data on the composition of the species and abundance of each species are necessary, since the respiration rate of these eukaryotic organisms is a function of their mass, which differs greatly among different species. Having this information and taking into account the temperature, on the basis of formula (2), we calculated the intensity of oxygen consumption and CO_2_ emissions by this group of activated sludge. Assuming that CO_2_ during the purification process is emitted as a result of biological processes and therefore, only by prokaryotes and eukaryotes, and knowing both the total amount of CO_2_ emitted and the amount of CO_2_ emitted by eukaryotes, we could evaluate the contribution of these two groups of organisms to the total production of CO_2_. The seasonal changes are shown in (Fig. 1).

For eukaryotes, all seasons are characterized by significant differences. The season with the highest CO_2_ emissions was summer, with about 0.7 × 10^−5^·g·m^−2^·min^−1^, followed by autumn 0.3 × 10^−5^·g·m^−2^·min^−1^, spring 0.2 × 10^−5^·g·m^−2^·min^−1^ and winter 0.1 × 10^−5^·g·m^−2^·min^−1^.

On the other hand, in the case of prokaryotes, there were no significant differences between seasons. The season with the highest emissions was autumn (1.3 g·m^−2^·min^−1^), followed by summer (1.2 g·m^−2^·min^−1^), spring (1.0 g·m^−2^·min^−1^) and winter (0.93 g·m^−2^·min^−1^). It should be noted that statistically significant differences in CO_2_ emissions between the seasons were observed for the eukaryotes (*p* ≤ 0.05) but not for prokaryotes.

**Figure 2:**
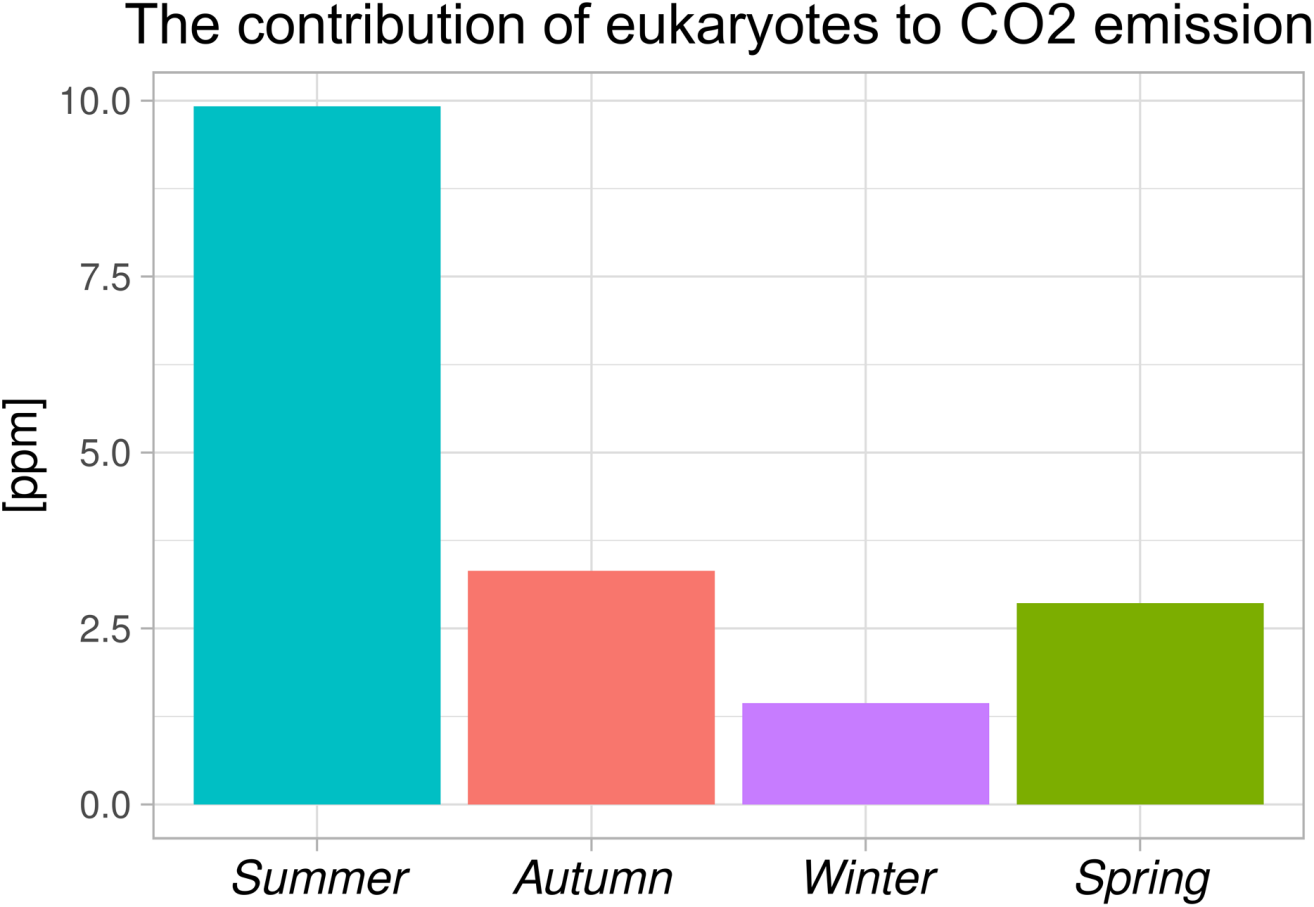
Share of eukaryotic organisms in total CO_2_ emissions during the aeration phase.

The contribution made by the eukaryotic organisms to the total CO_2_ emissions during the aeration phase in subsequent seasons is shown in Fig. 2. The contribution to the total CO_2_ emissions made by eukaryotic organisms during the aeration phase by season is shown in Fig. 2. The season with the highest contribution from eukaryotes was summer (20 °C) with almost 10 ppm. Next were autumn and spring (both 15 °C) with about 3.3 and 2.9 ppm respectively. In winter, the season with the lowest temperature (10 °C), the lowest contribution of CO_2_ emissions by eukaryotes was recorded. This suggests that these differences can probably be best explained by temperature.

## Discussion

It can be seen from Fig. 1A that CO_2_ emissions produced by the prokaryotes differed by season, (although differences were not statistically significant). As expected, the highest level was recorded in the summer and autumn seasons. The required temperature for most pollutant removal processes is considered to be higher than 15 °C for activated sludge (Keefer, 1962; Krishna & Van Loosdrecht, 1999; Henze et al., 2006) ); thus higher temperatures in summer and autumn may have caused higher microbial activity. The highest activity in the autumn season can be likely explained by two factors: the abundance of species and individuals of species after the summer season and the higher concentrations of nutrients in wastewater, related to inhabitants’ return from holiday. Many large cities in Poland where schools and colleges are based, are characterized by clearly visible variability/difference in wastewater inflow—both the quantity and the load of biogens. Between the holiday season and the winter semester, in Lublin, where approximately 40% of the population are non-resident pupils and students, there is a sharp increase in the amount of wastewater at the end of the holidays and return to school (September and October)—a change of around 46,000 to around 72,000 m^3^∙d^−1^. At the same time, such an increase in the amount of wastewater causes not only the inflow of a larger load of biogens—containing pollutants generated at that time—but also the flushing of sediment deposits, accumulating on the bottom of the gravitational sewer network during flows at a lower velocity. This velocity in holiday months (July and August) often decreases below the velocity of the hydraulic self-cleaning of channels in many places on the wastewater network. The only bigger and long lasting shift in quantity and quality of wastewater within the WWTP analysed, is noted during the summer in July and August (Jaromin, Szaja & Łagód, 2012).

In natural water bodies at temperatures below 10 °C, the intensity of biochemical processes decreases significantly, which is also manifested in a decrease in the formation of gases by microorganisms and other hydrobionts (Brylinsky & Mann, 1973; Sun et al., 2017).

In conditions of wastewater treatment facilities where the temperature even in winter does not drop below 10 °C and the inflow of organic substances (energy subsidies) does not stop, differences in CO_2_ emissions between seasons were not statistically significant (*p* > 0.05), what is important in terms of their influence onto atmosphere composition.

Discussion of the results is based on the available data, which can only be compared in an indirect way. The authors are not familiar with any publications reporting data for laboratory-scale SBR, which consider emissions from prokaryotes and eukaryotes and differences by season. Therefore, only total CO_2_ emissions were compared. Bao, Sun & Sun (2015) report that the annual average value of emissions from SBR chambers (in full-scale WWTPs) is about 2.8 g·m^−2^·min^−1^, which is more than twice that from the laboratory-scale SBR (1.12 g·m^−2^·min^−1^).

Additionally, our studies refer to the aerobic phase and therefore the results should be compared with the literature data for aerobic WWTP chambers. Yan, Li & Liu (2014) report the average CO_2_ emission fluxes in the aerobic areas of the reversed A2O (anoxic/anaerobic/aerobic) process and A2O (anaerobic/anoxic/aerobic) process were 0.7 and 1.14 g·m^−2^·min^−1^ respectively (both in the full-scale WWTP). This is similar to the results obtained by the authors for the laboratory-scale SBR. Furthermore, (Yan, Li & Liu, 2014) present the results for nine months throughout the year (from March to November) however, analysing their results in terms of seasonality, it is difficult to notice significant differences between subsequent seasons, similar to the presented in our work.

Significantly larger differences in CO_2_ production (*p* ≤ 0.05) were observed in the case of the eukaryotic organisms (Fig. 1B). The results from this study thus indicate that eukaryotes reacted more strongly to temperature changes than prokaryotes. The temperature seemed to have had a substantially greater influence here, as CO_2_ emissions in the summer were nearly three times greater than those in the autumn. The slightly higher emissions in the autumn (compared with the spring when the temperature was the same as during the autumn) can be explained in the same way as in the case of the prokaryotes—the favourable conditions prevailing during the Summer season and the larger load of pollutants in raw wastewater had an influence on the structure of the activated sludge in the autumn. As in the case of prokaryotes, the activity of the eukaryotes was lowest in the winter period.

As could be expected, the contribution of eukaryotic organisms to the total CO_2_ emissions is very small—at a level of several ppm (Fig. 2). The main role of eukaryotic organisms in the process of wastewater purification, is not to remove dissolved organic matter (this is the main role of the prokaryote), that is, a process responsible for the majority of CO_2_ emissions, as eukaryotes are predominantly bacteriophages. Eukaryotic organisms in activated sludge mainly have the function of regulators, maintaining bacterial communities in the phase of exponential growth and helping to form flocks with proper size, shape and density parameters (Čech, Hartman & Macek, 1994).

The results presented in this study should be treated as an estimation of the order of magnitude, rather than the exact evaluation, mainly because the investigations were carried out in the laboratory model. This process carried out on an industrial scale can be slightly different. It should be noted that scaling in SBR technology is a common problem in many investigations, as this kind of WWTP has a very wide range of applications: from a plant of several cubic meters in a small housing estate or a small production facility to the order of hundreds of thousands of cubic metres (Neczaj et al., 2008; Marañón et al., 2008; Gürtekin, 2014; Bao, Sun & Sun, 2016).

The uncertainty of our results should be also discussed. We found two major factors that could influence the results obtained: calculation of the eukaryotic organisms and estimation of CO_2_ emissions, produced by individuals of the given species. The error in the calculation of individuals of the species can primarily be caused by an unrepresentative subsample. To prevent this, the samples were taken in different places and counting was repeated. Moreover, the same operator counted the eukaryotes throughout the experiment. To minimize the human factor, the operator was rested and the light was optimized.

As shown in the Methods chapter, the calculation of CO_2_ emissions (caused by one individual) was based on the assumption that the amount of CO_2_ emitted is equivalent to the amount of oxygen consumed, which is burdened with an error that is, difficult to estimate. Despite all these sources of uncertainty, emissions caused by eukaryotic organisms at a level a few or several dozen ppm can be expected.

## Conclusions

To the best of our knowledge, the quantitative contribution of CO_2_ production by different components of activated sludge in WWTPs, has not been estimated to date. For eukaryotes, the season with the highest CO_2_ emissions was summer with about 0.7 × 10^−5^ g·m^−2^·min^−1^ and the lowest was winter, 0.1 × 10^−5^ g·m^−2^·min^−1^. In the case of prokaryotes, the differences between seasons were not significant. The contribution of eukaryotes to the total CO_2_ emissions is of the order of several or more ppm. The exact value is impossible to estimate because of seasonal changes. The vast majority of CO_2_ emissions is caused by prokaryotes. This is consistent with the fact that prokaryotes are responsible for wastewater purification from dissolved C compounds.

## Supplemental Information

10.7717/peerj.9325/supp-1 Supplemental Information 1 Raw data. Emission of CO_2_ caused by the activity of prokaryotes and eukaryotes in the subsequent seasons. These data were used for statistical analysis to compare emission in each seasons and to estimate the share of eukaryotic organisms in total CO_2_ emissions during the aeration phase.

## Additional Information and Declarations

### Data Availability

The following information was supplied regarding data availability: The raw data and measurements are available as a Supplemental File.
